# A Prospective Study on the Clinical Spectrum and Outcomes of Obstetric Critical Care Admissions at Konaseema Institute of Medical Sciences, Amalapuram - A Tertiary Care Centre in Coastal India

**DOI:** 10.7759/cureus.86661

**Published:** 2025-06-24

**Authors:** Varada A Hasamnis, Sri Vyjayanthi Kalyanapu, Laxmi Praveena Boddu, Palla Lydia Stotramani, Sravanthi Pathapati, Anusha Kota

**Affiliations:** 1 Department of Obstetrics and Gynaecology, Konaseema Institute of Medical Sciences and Research Foundation, Amalapuram, IND; 2 Department of Obstetrics and Gynaecology, Royal Lancaster Infirmary, Lancashire, GBR

**Keywords:** hypertensive disorders, intensive care in pregnancy, maternal mortality, obstetric critical care, postpartum hemorrhage, ruptured ectopic pregnancy, tertiary referral

## Abstract

Background: Managing critically ill obstetric patients is complex due to dual maternal and fetal considerations. In resource-limited settings, delayed referrals from peripheral centres often worsen outcomes.

Objective: To analyse the clinical spectrum, management, and outcomes of obstetric patients requiring critical care at a tertiary facility in coastal India.

Methods: A prospective observational study was conducted over two years, involving obstetric patients admitted to the critical care unit (CCU). Data on demographics, diagnoses, interventions, and outcomes were analysed descriptively.

Results: Most patients were unregistered and referred from peripheral centres. Obstetric haemorrhage and hypertensive disorders were the predominant causes of admission. Respiratory distress was a leading clinical indication. All maternal deaths occurred among referred cases. A considerable number of neonates required NICU care, indicating associated fetal compromise.

Conclusion: Strengthening antenatal care, timely referral, and implementing tools like the Modified Early Obstetric Warning Score (MEOWS) can improve maternal and fetal outcomes in critical obstetric cases.

## Introduction

Pregnancy is a dynamic physiological state that introduces significant anatomical, biochemical, and immunological changes [1,2]. While most pregnancies follow a normal course, a subset of women may develop life-threatening complications requiring intensive care support. The dual responsibility of managing both maternal and fetal well-being presents unique challenges to clinicians, particularly when complications arise suddenly or are referred late from peripheral facilities. Coastal Andhra Pradesh, where this study was conducted, includes semi-urban and rural areas with limited emergency transport infrastructure, variable health literacy, and suboptimal antenatal care coverage in certain districts. Many peripheral health centres lack intensive care capacity and advanced obstetric services, contributing to delays in stabilisation and referral. These regional constraints significantly influence the caseload at tertiary centres like some nearby medical colleges or centres, which often receive critically ill patients in advanced stages of obstetric complications [3,4].

Critically ill obstetric patients often present with a spectrum of emergencies, including severe haemorrhage, hypertensive disorders, sepsis, and medical conditions exacerbated by pregnancy. The management of such cases requires a multidisciplinary approach involving obstetricians, anaesthesiologists, intensivists, and neonatologists. Despite advancements in maternal healthcare, obstetric morbidity and mortality remain significant in low- and middle-income countries due to delayed referrals, lack of antenatal care, and insufficient resources at primary care levels [5-7].

In India, the maternal mortality ratio (MMR) has declined considerably from 130 to 97 per 100,000 live births in recent years, reflecting progress in healthcare infrastructure and policy initiatives [8]. However, preventable causes like postpartum haemorrhage and preeclampsia continue to contribute to maternal deaths, especially in rural and underserved regions. Tertiary care hospitals play a pivotal role in managing high-risk obstetric cases, often receiving referrals from peripheral health centres with minimal stabilisation.

This study was conducted to examine the clinical spectrum, causes, interventions, and outcomes associated with obstetric critical care admissions at a tertiary care centre in coastal India. By analysing the pattern and nature of such admissions, the study aims to identify gaps in referral practices and management, thereby providing insights for improving maternal and fetal outcomes through timely interventions and better preparedness.

## Materials and methods

Study design and setting

This was a hospital-based, prospective, observational study conducted at the Critical Care Unit (CCU) and the Department of Obstetrics and Gynaecology [9] at Konaseema Institute of Medical Sciences & Research Foundation, Amalapuram, a tertiary care teaching hospital in coastal India. The study covered a period of two years, from November 2022 to October 2024.

Study population

The study included obstetric patients who required critical care admission during pregnancy, labour, or within 42 days postpartum. These patients were admitted to the CCU from various departments, including the emergency room, labour room, operating theatre, and high-dependency units within the hospital.

Inclusion and exclusion criteria

All obstetric patients admitted to the CCU during pregnancy, labour, or within 42 days postpartum were eligible for inclusion in the study. Both direct admissions and referrals from peripheral health centres were considered, provided they met the obstetric-related criteria and gave informed consent. Women who were more than 42 days postpartum at the time of admission were excluded from the study. Additionally, cases unrelated to obstetric causes, such as accidental poisoning, and patients who were either unwilling to participate or had incomplete medical records, were also excluded.

Sample size and data collection

This was a descriptive observational study, and the sample size was not predetermined by statistical calculation. Instead, it included all obstetric patients who met the inclusion criteria and were admitted to the CCU over the two-year study period (November 2022 to October 2024). A total of 46 obstetric patients admitted to the CCU were included in the study. Purposive sampling was used to select cases based on inclusion criteria. Data were collected from patient admissions using structured proformas, clinical charts, and care logs maintained in real time during the study period by the Department of Obstetrics & Gynaecology and the CCU. Information included demographics (age, parity, antenatal registration), timing of admission (antepartum, postpartum), primary diagnosis (obstetric or medical), indications for CCU admission, comorbidities and interventions administered, and maternal and fetal outcomes. Although referral delay was not recorded consistently in our dataset, previous studies from South India, including Andhra Pradesh, report average delays of six to 12 hours, often due to transport constraints, delayed recognition, and under-equipped peripheral centres. Given the rural base of most referrals in this study, similar timeframes are likely [10].

Data analysis

All collected data were tabulated using Microsoft Excel (Microsoft Corporation, Redmond, WA) and analysed using SPSS version 20 (IBM Corp., Armonk, NY). Only descriptive statistics, such as frequencies and percentages, were applied. No inferential statistical tests were performed, and the analysis focused on summarising demographic variables, diagnostic categories, clinical interventions, and maternal and fetal outcomes.

Ethical considerations

The study received approval from the Institutional Ethics Committee of Konaseema Institute of Medical Sciences and Research Foundation, Amalapuram, under serial number IEC/PR/2022: 13/1/02.10.2022. Written informed consent was obtained from all participants or their legally authorised representatives, as applicable. Strict measures were taken to maintain patient confidentiality and ensure data privacy throughout the study.

## Results

Demographic and admission characteristics

A total of 46 critically ill obstetric patients were admitted to the CCU over the two-year study period. The majority were referred from peripheral health centres and were unregistered at the tertiary care centre (45/46; 97.82%), while only one patient (1/46; 2.17%) was registered antenatally. The most common age group was 20-30 years, comprising 29 patients (29/46; 63.04%), followed by nine patients aged 31-35 years (9/46; 19.56%), and three patients over 35 years (3/46; 6.52%). Adolescents under 20 years accounted for five cases (5/46; 10.86%).

**Figure 1:**
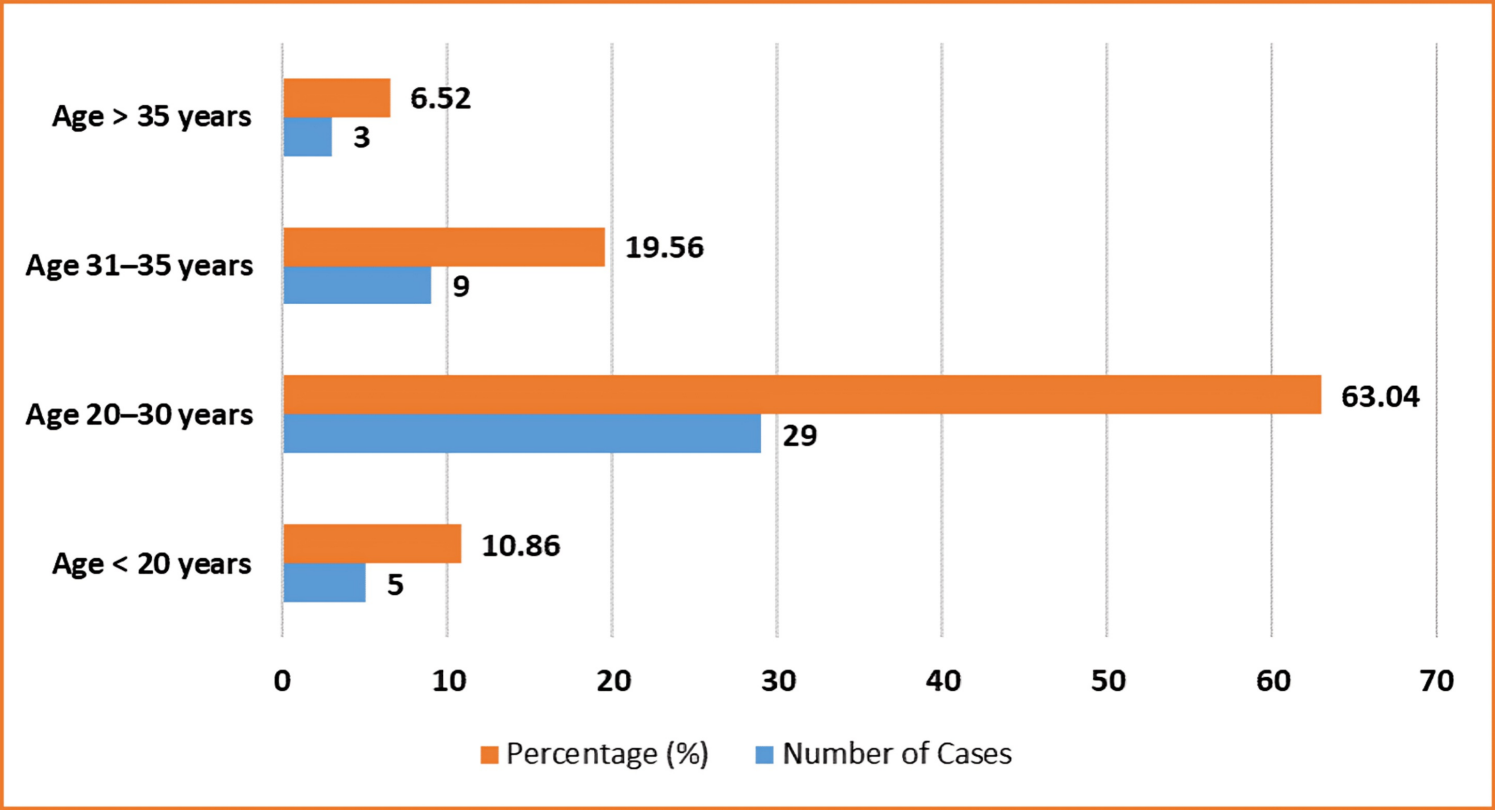
Age distribution of critically ill obstetric patients. X-axis: age groups (<20, 20–30, 31–35, >35); Y-axis: number of patients and percentages.

Antenatal (antepartum) admissions constituted 31 of the cases (31/46; 67.39%), while 15 patients (15/46; 32.6%) were admitted during the postpartum period. Regarding parity, 19 patients were nulliparous (19/46; 41.3%), 14 were first para (14/46; 30.43%), 12 were second para (12/46; 26.08%), and only one patient (1/46; 2.17%) had a parity of three or more (Table 1 and Figure 1).

**Table 1:** Demographic and admission characteristics of critically ill obstetric patients (n = 46). Parity refers to the number of pregnancies reaching ≥20 weeks of gestation. Nulliparous = no prior births; first para = one birth; second para = two births; third para or more = ≥3 births.

Parameter	Number of cases	Percentage (%)
Antenatal care - registered	1	2.17
Antenatal care - unregistered	45	97.82
Age < 20 years	5	10.86
Age 20–30 years	29	63.04
Age 31–35 years	9	19.56
Age > 35 years	3	6.52
Antepartum admissions	31	67.39
Postpartum admissions	15	32.6
Nulliparous	19	41.3
First para	14	30.43
Second para	12	26.08
Third para or more	1	2.17

Diagnosis and indications for critical care admission

Among the 46 admissions, 30 cases (30/46; 65.21%) were due to direct obstetric causes, while 16 cases (16/46; 34.78%) were attributed to medical conditions complicating pregnancy. The leading obstetric cause was ruptured ectopic pregnancy, observed in 14 patients (14/30; 46.66%), followed by postpartum haemorrhage in eight patients (8/30; 26.66%) and hypertensive disorders, including severe preeclampsia and eclampsia, in seven cases (7/30; 23.32%). Septic abortion accounted for one case (1/30; 3.33%).

Among the 16 medical complications, cardiac disorders such as rheumatic heart disease, cardiomyopathy, and pulmonary arterial hypertension were present in 11 patients (11/16; 68.75%). Liver-related conditions, including acute fatty liver of pregnancy, jaundice, and hepatic failure, occurred in three patients (3/16; 18.75%), while respiratory disorders and pyrexia with thrombocytopenia were each noted in one patient (1/16; 6.25%).

**Figure 2:**
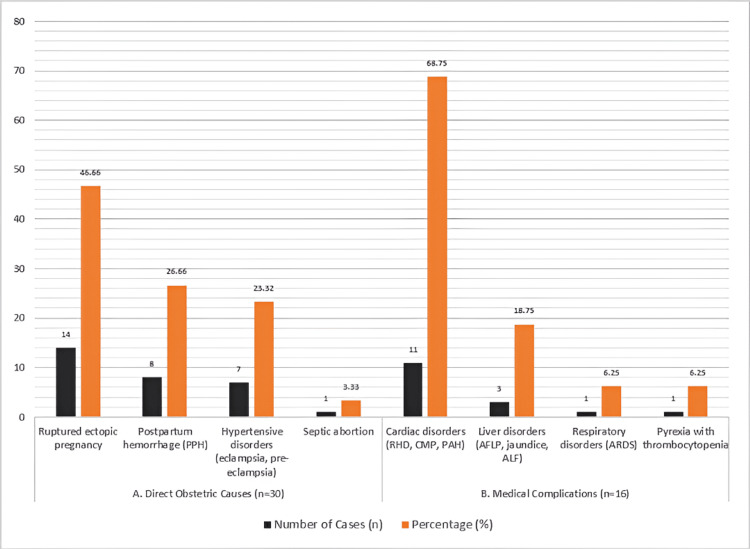
Distribution of admission of (A) direct obstetric causes (n = 30) and (B) medical complications (n = 16). X-axis: number and percentage of the cases; Y-axis: cause category. RHD: rheumatic heart disease; CMP: cardiomyopathy; PAH: pulmonary arterial hypertension; AFLP: acute fatty liver of pregnancy; ALF: acute liver failure; ARDS: acute respiratory distress syndrome.

The most common clinical indication for CCU admission was respiratory distress, reported in 10 patients (10/46; 21.7%). This was followed by low Glasgow Coma Scale (GCS) scores in nine patients (9/46; 19.56%), hypotension and oliguria each in eight patients (8/46; 17.4%), disseminated intravascular coagulation (DIC) in seven patients (7/46; 15.21%), refractory seizures in three patients (3/46; 6.52%), and maternal collapse in one patient (1/46; 2.17%) (Table 2 and Figure 2).

**Table 2:** Indications for critical care unit (CCU) admission.

Diagnosis/indication (indications for CCU admission)	Number of cases	Percentage (%)
Respiratory distress	10	21.7
Low Glasgow Coma Scale (GCS) score	9	19.56
Hypotension	8	17.4
Oliguria	8	17.4
Disseminated intravascular coagulation	7	15.21
Refractory seizures	3	6.52
Maternal collapse	1	2.17

Interventions and comorbidities

Among the 46 critically ill obstetric patients, blood transfusion was the most frequent intervention, administered to 25 patients (25/46; 54.34%). Advanced life support was provided in 20 cases (20/46; 43.47%), while inotropic support was required in 19 cases (19/46; 41.3%). Central venous line insertion was performed in 16 patients (16/46; 34.78%). Pharmacological interventions included administration of antihypertensive agents in 13 cases (13/46; 28.26%), anticonvulsants in eight cases (8/46; 17.39%), and renal dialysis in eight cases (8/46; 17.39%).

Comorbidities were identified in 12 patients (12/46; 26.08%). Among these, cardiac diseases were the most common, present in five patients (5/12; 41.66%). Anaemia, hypothyroidism, and pregnancy-induced hypertension were each observed in two patients (2/12; 16.66%), while epilepsy was noted in one case (1/12; 8.3%) (Table 3).

**Table 3:** Interventions and comorbidities.

Intervention/comorbidity	Number of cases	Percentage (%)
Interventions		
Blood transfusion	25	54.34
Advanced life support	20	43.47
Inotropic drugs	19	41.3
Central line	16	34.78
Antihypertensives	13	28.26
Anticonvulsants	8	17.39
Dialysis	8	17.39
Comorbidities (n = 12)		
Cardiac disease	5	41.66
Anaemia	2	16.66
Hypothyroidism	2	16.66
Pregnancy-induced hypertension	2	16.66
Epilepsy	1	8.3

Obstetric and maternal outcomes

Among the 46 patients, caesarean section was the most common mode of delivery, performed in 20 cases (20/46; 43.47%). Exploratory laparotomy for ruptured ectopic pregnancy was carried out in 14 patients (14/46; 30.43%), while vaginal delivery occurred in eight patients (8/46; 17.39%). Abortion was reported in three cases (3/46; 6.52%), and one patient (1/46; 2.17%) remained undelivered due to maternal death prior to delivery.

**Figure 3:**
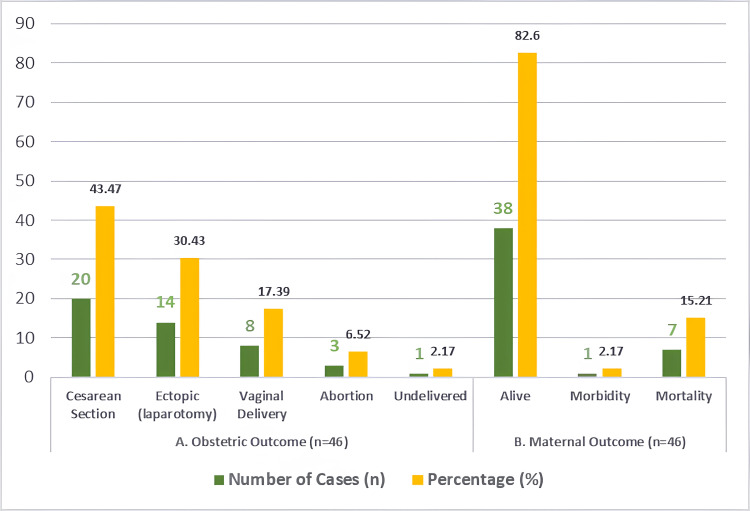
Distribution of (A) obstetric and (B) maternal outcomes among critically ill obstetric patients. Values are shown as numbers and percentages in each section.

The maternal survival rate was 82.6%, with 38 patients surviving (38/46), while one patient (1/46; 2.17%) experienced long-term morbidity. There were seven maternal deaths (7/46; 15.21%), all of which occurred among patients referred from peripheral centres. Notably, although hypertensive disorders were less common than obstetric haemorrhage, they contributed disproportionately to maternal mortality (Figure 3).

Fetal outcomes

**Figure 4:**
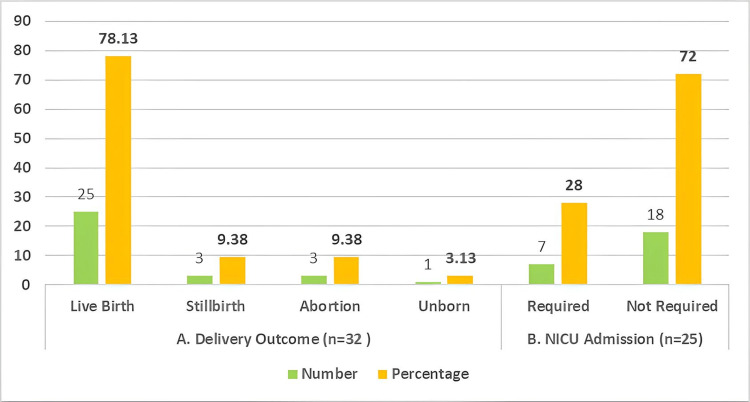
(A) Fetal outcomes and (B) NICU admission.

Out of the 32 pregnancies that progressed to delivery, 25 resulted in live births (25/32; 78.13%). Stillbirths and abortions were each reported in three cases (3/32; 9.38%), and one fetus (1/32; 3.13%) remained unborn due to maternal demise. Among the 25 live-born neonates, seven required NICU admission (7/25; 28%), while 18 did not require intensive care (18/25; 72%) (Figure 4).

## Discussion

This study explored the clinical characteristics, underlying causes, interventions, and outcomes of critically ill obstetric patients admitted to a tertiary care critical care unit. The results reflect the ongoing challenges in managing obstetric emergencies, particularly in resource-limited settings where delayed referrals from peripheral centres are common.

A large proportion of patients were unregistered and referred from primary or secondary care facilities, underscoring major gaps in antenatal care and early risk identification. This pattern aligns with previous observations of high dependency on tertiary referrals due to insufficient infrastructure and training at peripheral levels [10,11]. Similar patterns have been reported in other Indian cohorts, where delayed referrals and direct obstetric complications like haemorrhage and hypertensive disorders were predominant causes of ICU admission [12,13]. A study postulated that at a dedicated obstetric ICU, the majority of ICU admissions were referrals, and a significant proportion were un-booked, highlighting persistent deficiencies in early obstetric risk stratification and escalation of care [14-17].

Most patients fell within the 20-30 year reproductive age range, similar to findings from other studies evaluating critically ill obstetric cohorts in both regional and international contexts [10,18]. A predominance of antepartum admissions indicates that life-threatening complications typically manifest during the later stages of pregnancy, when the physiological burden is greatest. This is supported by findings of a study that reported that over two-thirds of admissions occurred during the third trimester, often due to late-onset hypertensive or haemorrhagic events [19].

Direct obstetric causes, such as ruptured ectopic pregnancy and postpartum haemorrhage, were the most common reasons for admission. Despite haemorrhage being more frequent, hypertensive disorders were associated with a disproportionately higher maternal mortality rate, suggesting a more subtle clinical progression and greater risk of delayed diagnosis and intervention [13,20]. This trend was also observed by a study, which noted that while obstetric haemorrhage accounted for the majority of admissions, eclampsia and preeclampsia were independently associated with poorer prognoses and higher ICU resource utilisation [21,22].

Medical complications, particularly cardiac disorders, contributed significantly to the total maternal deaths observed. These findings reinforce the growing recognition of non-obstetric medical conditions as key contributors to morbidity and mortality in pregnancy, a trend echoed in recent literature on obstetric critical care in South Asia [12,13].

Intervention requirements such as blood transfusion and ventilatory support were frequent, indicating the need for rapid stabilisation protocols and multidisciplinary management. The overall maternal survival rate was encouraging; however, the 15.21% (7/46) mortality rate among referred patients highlights the critical impact of delayed care. Similar findings have been documented in other Indian cohorts, where referral-related delays were a statistically significant factor in adverse outcomes [23]. For instance, a similar study demonstrated that more than 80% of patients who died had received inadequate pre-referral stabilisation, emphasising the need for structured triage and referral training at the primary health centre/community health centre level [24].

Fetal outcomes revealed that although maternal survival improved with prompt intervention, a significant proportion of neonates required intensive care. This suggests ongoing fetal compromise, particularly in the context of delayed maternal stabilisation [13]. The prospective design of this study minimised the risk of missing data and allowed for real-time recording of clinical interventions and patient outcomes, thereby enhancing data accuracy and reliability. A prominent study found that NICU admissions were nearly twice as frequent among neonates delivered to mothers who had undergone emergency obstetric transfers, reinforcing the fetal burden of maternal instability on arrival [25].

The prospective design of this study minimised the risk of missing data and allowed for real-time recording of clinical interventions and patient outcomes, thereby enhancing data accuracy and reliability. These findings collectively underscore the importance of strengthening primary and secondary health systems. Implementing tools such as the Modified Early Obstetric Warning System (MEOWS) and ensuring timely referral and transport can significantly reduce delays. Structured referral protocols, community awareness, and continued training for healthcare providers are essential to reduce preventable maternal and fetal morbidity and mortality in high-risk obstetric cases [20,26].

This study has certain limitations. Being a single-centre prospective study with a limited sample size, it relied on the accuracy and completeness of existing records, which may have led to missing or underreported variables. Conducted at a single tertiary care centre, the findings may not be generalizable to other regions. Additionally, the small sample size of critically ill obstetric cases may limit the statistical power of the analysis. Long-term maternal and neonatal outcomes were not assessed, as follow-up data beyond hospital discharge were unavailable. This limits the ability to evaluate post-discharge complications, maternal readmissions, or neurodevelopmental outcomes in neonates. Future studies should incorporate structured follow-up to assess the extended trajectory of recovery and neonatal well-being.

## Conclusions

Obstetric haemorrhage and hypertensive disorders remain the leading causes of critical care admissions among pregnant women. A significant proportion of these patients were unregistered and referred late from peripheral centres, contributing to adverse maternal and fetal outcomes. Timely recognition, early referral, and a multidisciplinary management approach are crucial to improving survival. Strengthening peripheral health infrastructure and ensuring the availability of skilled personnel remain essential. To address systemic delays, policy-level strategies such as mandatory risk stratification protocols during antenatal visits, deployment of mobile ICU support teams, and regular obstetric emergency drills at peripheral health centres should be considered. Implementing tools like the MEOWS can further enhance early detection and reduce delays. Enhancing community awareness and refining structured referral protocols may significantly lower maternal mortality, ultimately improving the overall quality of obstetric care in resource-limited settings.
